# Impacts of Protease Sources on Growth and Carcass Response, Gut Health, Nutrient Digestibility, and Cecal Microbiota Profiles in Broilers Fed Poultry-by-Product-Meal-Based Diets

**DOI:** 10.3390/metabo15070445

**Published:** 2025-07-02

**Authors:** Muhammad Shahbaz Zafar, Shafqat Nawaz Qaisrani, Saima, Zafar Hayat, Kashif Nauman

**Affiliations:** 1 Department of Animal Nutrition, Faculty of Animal Production & Technology, University of Veterinary & Animal Sciences, Lahore 54000, Pakistan; shahbaz.zafar@uvas.edu.pk (M.S.Z.); saima.mahad@uvas.edu.pk (S.); 2 Department of Animal Sciences, College of Agriculture, University of Sargodha, Sargodha 40100, Pakistan; zafarhayat@uos.edu.pk; 3 Department of Meat Science & Technology, Faculty of Animal Production & Technology, University of Veterinary & Animal Sciences, Lahore 54000, Pakistan; kashif.nauman@uvas.edu.pk

**Keywords:** protease, growth performance, gut health, amino acids digestibility, broiler

## Abstract

Background: The current study aimed to evaluate the effects of the supplementation of protease sources on growth and carcass response, gut health, nutrient digestibility, and cecal microbiota profiles in broilers fed poultry-by-product-meal (PBM)-containing diets. Methods: In total, 800 one-day-old mixed-sex broilers (Arbor Acres) were weighed and allocated to one of the four dietary treatments in a completely randomized design, with eight replicates and 25 birds each per replicate. The treatments were as follows: (1) T_0_, control diet (without protease supplementation and 3% PBM); (2) T_1_, control diet supplemented with acidic protease at 100 g/ton (50,000 U/g); (3) T_2_, control diet supplemented with alkaline protease at 200 g/ton (25,000 U/g); (4) T_3_, control diet supplemented with neutral protease at 200 g/ton (25,000 U/g). Results: Protease supplementation enhanced (*p* < 0.05) body weight gain and the feed conversion ratio, predominantly in broilers fed PBM-based diets containing alkaline protease. Alkaline protease supplementation increased (*p* < 0.05) the apparent ileal digestibility of proteins (AIDP) by 4.3% and the apparent ileal digestibility of amino acids (AIDAA) by up to 5.8%, except for ornithine. Increments (*p* < 0.05) in carcass, breast, and leg quarter yields due to protease supplementation were evident, particularly in broilers fed diets containing alkaline protease. Alkaline protease improved (*p* < 0.05) the duodenal villus height (VH), reduced the crypt depth (CD), and increased the villus height to crypt depth ratio (VCR). Alkaline protease supplementation reduced (*p* < 0.05) cecal counts of *Salmonella*, *Escherichia coli*, and *Clostridium* in the broilers, whereas it increased (*p* < 0.05) the *Lactobacillus* counts. Conclusions: the supplemented alkaline protease resulted in improved growth performance and carcass traits, better gut health, as well as improved ileal digestibility of nutrients, including crude protein (CP) and acid insoluble ash (AIA), with a more balanced cecal microbial composition in broilers.

## 1. Introduction

Soybean meal (SBM) is the primary and most commonly used plant protein feedstuff in poultry diets. However, various factors, including high cost, perceptions of consumers regarding genetically modified feedstuffs, comparatively inadequate SBM supply to non-growing countries, and environmental concerns drive the exploration of alternative potential protein feedstuffs for broilers. Potential alternatives to SBM include canola meal (CM), rapeseed meal (RSM), sunflower meal (SFM), guar meal (GM), and poultry by-product meal (PBM) [1]. These alternatives can at least partially reduce dependence on SBM and help to bridge the gap between supply and demand for conventional feedstuffs in poultry production [2]. PBM is an animal protein by-product produced via a rendering process with high (58.4–62%) crude protein (CP) [3]. Using PBM in poultry feeds, therefore, reduces reliance on SBM and encourages sustainability by decreasing poultry industry waste. Among the factors affecting the digestibility of animal protein ingredients for broilers are the impact of thermal processing and the type of carcass components used in the process [4], which limit its utilization in broiler feeds [5].

The poor digestibility of animal protein sources, potentially used in broiler diets, can be improved in various ways, including the use of enzyme supplementation, such as protease, to break down protein more effectively; the inclusion of organic acids to lower GIT pH; promoting better enzyme activity and the addition of probiotics, which enhance gut microbiota balance; and supporting overall nutrient absorption and protein digestibility [6]. Proteases can potentially enhance growth performance in poultry, as the activity of pancreatic protease is initially low at hatching and gradually increases up to 21 d of age. Due to the underdeveloped digestive system of chicks, digestive proteases may limit protein digestion in young birds [3]. The CP and amino acid (AA) digestibility documented for poultry, however, suggests that a significant amount of protein remains undigested as it passes through the GIT [7]. This undigested protein calls for the use of exogenous proteases in broiler diets to increase the digestibility of protein by enhancing pancreatic protease activity and breaking down resistant proteins into smaller peptides. Additionally, these exogenous proteases help to reduce the quantity of undigested protein passing the hindgut, leading to better growth, reduced protein waste, and a healthier gut environment. Exogenous enzymes have been widely used to enhance diet uniformity, specifically when the variability of ingredients is considered to be a factor that limits the predictability of broiler performance [3].

Protease supplementation showed that digestibility was improved for all AAs [8] or some AAs (lysine, threonine, and cysteine) [9]. Protease supplementation improved the overall microbial population in the small intestine and the relative bacterial count, including of *Lactobacillus*, which is considered beneficial for health [10]. It is believed that protease supplements may influence the microbial population by altering the substrates available for microbes. The increased accessibility of AAs, for instance, was found to either promote or inhibit the growth of specific microbes [11]. Earlier studies on broilers and turkeys demonstrated that protease supplementation improved the villus height (VH) and villus height to crypt depth ratio (VCR), increased gut resilience by reducing inflammatory reactions, enhanced tight junction and mucin integrity [12], reduced intestinal *Clostridium* counts, and improved intestinal mucosal characteristics [13].

Some studies reported no effects [14,15] or negative impacts [16] of protease supplementation on nutrient digestibility. The influence of protease supplementation on CP and AA digestibility has been inconsistent. This inconsistency may be due to variations in the formulation of the test diet [17,18], supplementation level [19], or combined supplementation with other enzymes [20]. The divergent results could be due to the characteristics of the supplemented protease, as its efficacy can be influenced by environmental factors such as pH and temperature [3].

Keeping in view the scarce availability of the literature and inconsistent findings, the study aimed to evaluate the effect of protease sources (acidic, alkaline, and neutral) on growth and carcass response, gut health, nutrient digestibility, and cecal microbiota profiles in broilers fed a PBM-based diet. It was hypothesized that protease supplementation may affect AA digestibility and the microbial composition in the distal portion of the small intestine.

## 2. Materials and Methods

### 2.1. Animal Ethics

Experimental procedures were approved (No. DR/85; Dated: 15 November 2023) by the Animal Ethical Review Committee of the University of Veterinary and Animal Sciences, Lahore, Pakistan. Ethical approval was sought before the study.

### 2.2. Experimental Design, Birds, and Housing

In total, 800 mixed-sex Arbor Acres broiler hatchlings were allocated into 32 floor pens, each containing 25 birds (stocking density: 0.75 ft^2^/bird) and bedded on rice husk (3–4 inches) in a completely randomized design (CRD). Each pen had two feeders and 4 drinking nipples with cups. For the first 3 d, the lighting schedule was set at 23 h of light following 1 h of darkness, which was then effectively adjusted to 16 h of light and 8 h of darkness, with an LED lighting intensity of 20 lux at the level of the bird during the entire experiment. The ambient temperature of the shed was sustained at 34 °C with the help of an electric brooder, and then gradually reduced to a constant value of 22 °C and kept until the end of the trial. The birds were vaccinated against Newcastle disease on d 4 (intraocular) and d 20 (via drinking water), and against infectious bursal disease on d 8 (intraocular) and d 24 (via drinking water) according to the strain-specific recommendations. Feed and water were provided ad libitum throughout the trial (1 to 35 d of age).

### 2.3. Dietary Treatments

The study comprised 4 dietary treatments. The control diet (T_0_) had 3% PBM (comprised mainly of poultry carcass, including heads, feet, gizzards, intestines (without their contents), and undeveloped eggs, also excluding feathers) without protease supplementation (Table 1). For the other 3 treatments, the control diet was supplemented with 3 different proteases: T_1_, control diet plus acidic protease at 100 g/ton (50,000 U/g); T_2_, control diet plus 200 g/ton alkaline protease (25,000 U/g), and T_3_, control diet plus 200 g/ton neutral protease (25,000 U/g). The inclusion rates (g/ton) of protease varied between the treatments and the overall enzymatic activity per ton of feed was kept consistent. All of the protease-supplemented diets (T1–T3) provided 5,000,000 units of the enzyme per ton to ensure the functional equivalency of protease supplementation. The diets were iso-caloric, iso-nitrogenous, and formulated to meet or exceed the nutrient recommendations of Arbor Acres, and fed as a starter (1 to 10 d), grower (11 to 24 d), and finisher diet (25 to 35 d). For the digestibility trial, acid insoluble ash (1% Celite^®^) was used as an indigestible marker in the ration for the last 3 days of the trial.

### 2.4. Exogenous Proteases

Three protease sources (acidic, alkaline, and neutral; Suntaq International Ltd., Shenzhen, China) were used in this study. The acidic protease was from *Aspergillus niger*, and the alkaline and neutral proteases was from *Bacillus subtilis*. The activity of proteases was determined by following the method of Xu et al. (2003) [21].

### 2.5. Traits Measured

#### 2.5.1. Growth and Carcass Parameters

Production parameters, such as feed intake (FI), body weight gain (BWG), and feed conversion ratio (FCR), were documented stage-wise for the starter, grower, and finisher phases. The health, welfare, and mortality of broilers were observed daily. At the end of the experiment (d 35), 3 broilers from each pen (24 birds per treatment) were randomly selected, slaughtered using the halal method [22], and processed to determine carcass weight (excluding giblets, % of live weight (LW)), breast (BY), and leg quarter yield (LQY) weight (% of carcass), as adopted by Ojewola and Onwuka (2001) [23].

#### 2.5.2. Tissue Collection and Histo-Morphological Analysis of the Intestine

A two centimeter duodenal segment was collected from the mid-section to assess the duodenal morphology. After washing with cold normal saline (0.9%), the sample was instantly placed in sterile plastic containers with buffered formalin (10%) for transportation to the laboratory. Histological specimens were shifted to a 70% ethanol solution and processed through drying, clearing, and execution, fixed in paraffin, and cut into 5 μm sections. Six cross-sections per bird were prepared for histological analysis. The villus height (VH, the distance from the tip of the villus and the villus–crypt junction) and crypt depth (CD) (the distance from the junction to the basement membrane of the epithelial cells) were analyzed on 10 intact, well-aligned villi (from the collected sample) per broiler. This process was performed using a camera-fitted compound microscope, and ImageJ software (Fiji version) was used for the measurements.

### 2.6. Sample Analysis

For proximate (dry matter (DM), crude fiber (CF), ether extract (EE), CP, and crude ash) analysis, the ration and ileal contents were ground (0.5 mm) with a vibrating disk mill (Fritsch Pulverisette 14, Fritsch GmbH, Amberg, Germany) following the standard procedures of AOAC (2006) [24]. To measure the apparent ileal digestibility (AID) of nutrients (CP and AA), 3 broilers were randomly selected from each treatment and slaughtered. The ileal digesta were collected by gently pressing the region connecting the yolk sac with the distal portion of the ileum, at least 20 mm above the ileo-cecal joint of the slaughtered broilers. The collected digesta samples were transferred into a plastic container and then refrigerated thereafter at −20 °C till further analysis. The CP percentage in the ration and the ileal contents was calculated as N_2_% × 6.25, with N_2_ being measured via the Micro Kjeldahl method using a catalyst (CuSO_4_) (ISO 5983). The AID of CP and AAs was determined using the following equation [25]:

$$AID\;\left(\%\right)=\frac{\frac{CPd}{AiAd}-\frac{CPi}{AiAi}}{\frac{CPd}{AiAd}}\times 100$$

where CPd and AiAd are the CP and acid insoluble ash (AIA) contents in the ration, respectively, and CPi and AiAi are the same dietary contents in the ileal digesta, respectively.

The AA quantities in the ileal digesta were analyzed using the procedures of Ahmed et al. (2020) [26] with an AA analyzer (Biochrom 30^+^, Biochrom Limited. Cambridge, UK). Briefly, finely ground (up to 0.5 mm) samples were subjected to oxidation with performic acid to conserve methionine and cysteine. This process conserves these AAs as met—methionine sulphone and cys—cystic acid. The samples were hydrolyzed thereafter with 6 M of hydrochloric acid/phenol for 24 h and the pH was subsequently adjusted to 2.2. After filtration, the samples were transferred into sample vials for AA quantification using a Biochrom 30^+^ AA analyzer with ion exchange chromatography.

### 2.7. Cecal Microbiota

On d 35, 3 broilers from each pen (24 birds per treatment), as discussed earlier, were selected, slaughtered, and the cecal samples were transferred into aseptic bottles. The bottles were placed on ice and shipped to the laboratory. Bacterial counts were measured in the samples by making serial 10-fold dilutions (1% peptone solution) on selective agar media. Brilliant Green agar (Oxoid, Basingstoke, UK) was used for *Salmonella*, and samples were incubated for 24 h at 35 °C. For *E. coli*, MacConkey agar (Oxoid, Basingstoke, UK) was used, and the samples were incubated for 48 h at 35 °C. The Reinforced Clostridial agar (Oxoid, Basingstoke, UK) was used for *Clostridia*, and the samples were incubated for 24 h at 30 °C. Lastly, Man Rogosa and Sharpe (MRS) agar (Oxoid, Basingstoke, UK) was used for *Lactobacilli*, and the samples were incubated for 48 h at 35 °C. The detection of these bacteria was carried out based on their growth on specific media. The colonies of these bacteria were counted with the help of a colony counter. The microflora counts were represented as log_10_ colony-forming units per gram of the gut material.

### 2.8. Data Analysis

The data were analyzed using one-way ANOVA in PROC GLM of SAS (version 9.1; SAS Inst. Inc., Cary, NC, USA), and the means were compared using Duncan’s Multiple Range Test (DMR) based on the following statistical model:

$$Y_{ijk}=µ+ES_{i}+\varepsilon_{ijk}$$

where Y_ijk_ = dependent variables, μ = mean of the population, ES_i_ = effects of different protease sources, and e_ijk_ = residual effect.

Differences between treatments were considered significant at a probability level of 5% or lower.

## 3. Results

### 3.1. Growth Performance

The influence of protease supplementation on the growth performance (FI, BWG, and FCR) of the broilers is summarized in Table 2. The observations indicate that broilers consuming rations supplemented with protease had better (*p* < 0.05) BWG (starter and grower phases) and improved FCR in all phases in comparison with those fed the control diets. There was no significant difference in FI from d 1 to 35. Alkaline protease supplementation (T_2_) resulted in a 7.02% increased BWG and 7.21% improved FCR throughout the starter period. During the grower period, the T_2_ showed a 5.83% increase in BWG and a 7.04% improvement in FCR. During the finisher period (25 to 35 days), broilers fed the T_2_ diet had a 4.14% improvement in FCR. The overall results of the present research throughout the trial duration (1 to 35 d) showed that the broilers fed alkaline protease-supplemented diets had a 4.72% higher BWG and a 5.4% improved FCR in comparison the control group. Additionally, the improvements in performance parameters were notable (*p* < 0.05) in comparison with acidic and neutral protease-supplemented diets.

### 3.2. Carcass Characteristics

Table 3 shows the impact of protease supplementation on carcass traits in the broilers. Carcass yield was improved (*p* < 0.05) in the broilers consuming protease-supplemented PBM-based rations than those consuming the control diet. Broilers consuming rations supplemented with alkaline protease showed a 5.6% increase in DWG, a 6.1% improvement in DWWG, a 12.9% higher BY, and a 3.54% increase in LQY in comparison with the control group. The broilers fed diets containing neutral and acidic proteases had a 4.6 and 1.5% greater DWG, 3.4 and 1.92% higher DWWG, 7.2 and 2.4% higher BY, and 2.04 and 1.4% increased LQY, respectively, in comparison with those consuming the control diet.

### 3.3. Gut Health

The impacts of the test diets on the duodenal VH, crypt depth (CD), and their ratio (VCR) in broilers are summarized in Table 4. The findings indicate that protease supplementation improved (*p* < 0.01) the gut health parameter in comparison with the control diet. Broilers fed an alkaline protease-supplemented diet had a 10.9% increase in VH, a 9.8% decrease in CD, and a 22.8% improvement in the VCR compared to the control. On the other hand, broilers fed a neutral or acidic protease-supplemented diet had a 9.2 and 8.5% higher VH, 7.1% lower CD, and 17.1 and 16.5% better VCR than the control, respectively.

### 3.4. Apparent Ileal Digestibility of Crude Protein, Amino Acids, and Dry Matter

Table 5 demonstrates the effects of the test diets on AIDP, AIDAA, and DM in the broilers. The observed findings indicate that protease supplementation improved (*p* = 0.01) nutrient digestibility in comparison with the control diet. Broilers fed diets containing alkaline protease had a 4.3% increase in AIDP and a 3.4% to 5.8% increase in AIDAA. Other than that, broilers that were fed diets with neutral or acidic protease had a 2.4 and 1.7% higher AIDP and 2.2 to 3.6 and 1.3 to 2.4% higher levels of AIDAA compared to those that were fed the control diet. Also, the AID of certain amino acids, such as Leu, Arg, Ala, Meth, and His was higher (*p* = 0.01) in broilers that were consuming diets with protease compared to those fed the control diet.

### 3.5. Cecal Microbiota

Table 6 shows the effects of experimental rations on the composition of cecal microbiota in the broilers. The findings indicate better (*p* < 0.01) cecal microbiota composition (*Lactobacillus*, *E. coli*, *Salmonella,* and *Clostridium*) in broilers consuming rations containing PBM supplemented with protease in comparison with the control diet. The broilers fed a diet containing alkaline proteases showed an 18.5% increase in the cecal *Lactobacillus* count and a decrease of 37.9, 23.7, and 19.5% in the *E. coli*, *Salmonella*, and *Clostridium* counts, respectively. Conversely, the broilers fed diets containing neutral protease-supplemented diets had an 8.61% increase in the cecal *Lactobacillus* count, with reductions of 36.8, 23, and 8.95% in the *E. coli*, *Salmonella*, and *Clostridium* counts, respectively. The broilers consuming an acidic protease-supplemented diet showed a 4.4% increase in their cecal *Lactobacillus* counts and decreases of 28.1, 20.1, and 6.6% in their *E. coli*, *Salmonella*, and *Clostridium* counts, respectively, compared with those consuming the control diet.

## 4. Discussion

The present study aimed to investigate the impact of protease sources on the production performance and carcass traits, gut health, cecal microbiota profiles and the AID of CP and AAs in broilers fed PBM-based diets. It was hypothesized that the dietary supplementation of protease may improve the above mentioned parameters in the broilers. The improved duodenal VH and VCR and reduced CD were associated with a healthier gut.

Based on earlier findings [5,27] on various dietary concentrations of PBM in broiler diets, the observed improvement in performance aligns with these studies, in which FI remained unaffected by the inclusion of PBM at 3%. Moreover, protease supplementation also did not affect FI [19]. The improved zootechnical performance observed with protease supplementation, without affecting feed intake, suggests that the improvement in performance may be due to the improved digestibility of energy and AAs. The observed better zootechnical performance (BWG and FCR) after alkaline protease supplementation in the broiler is aligned with the reported findings [28,29], which may possibly be due to better nutrient digestibility leading to the improved growth efficiency of broilers [28]. This better zootechnical productivity observed in broilers fed protease-supplemented PBM-based diets could be related to improved intestinal health, higher activity of digestive enzymes, and the effective utilization of nutrients, particularly protein [15,19]. Consistent with these results, Angel et al. (2011) observed a 7.1% greater BWG and 5.2% improved FCR in broilers consuming alkaline protease-supplemented diets compared to a diet without protease [19]. Cowieson and Ravindran (2008) also reported an 8.8% improved BWG with a 6.2% improved FCR by supplementing an enzyme cocktail (xylanase, amylase and alkaline protease) in broilers [30]. Qiu et al. (2023) found that alkaline protease supplementation resulted in a 26.9% improved average daily gain (ADG) and 4.2% improved FCR in comparison with the control diet without protease [31]. Yi et al. (2024) observed a 3.1% increase in ADG in broilers fed alkaline protease-supplemented diets compared with the control group, while FCR remained unaffected [32]. This improved ADG could be due to the fact that alkaline proteases in the small intestine optimize protein digestion, resulting in the higher availability of AAs, which is critical for muscle development. Some studies, however, have shown that growth performance (BWG and FCR) in broilers was unaffected by protease supplementation [33]. The observed differences in the zootechnical performance of broilers by supplementing protease could be related to the variations in the feed formulation or source of the product.

The carcass yield in poultry is a reliable indicator of diet quality. The improved carcass traits (BY, LQ, DWG, and DWWG) observed in broilers consuming PBM-based diets supplemented with protease align with the expectations and are consistent with the findings reported in the published literature on broilers [34]. Hussain et al. (2019) reported a 3.3% increased BY in broilers fed alkaline protease-supplemented diets in comparison with those fed the control diet [35]. Li et al. (2023), likewise, found a 0.8% increased carcass and about 6% increased thigh muscle yield in broilers fed an alkaline protease-supplemented diet [36]. Qiu et al. (2023) reported a 0.4% higher dressing percentage and 2.9% improved leg quarter yield from alkaline protease supplementation in broilers [31]. The increased carcass yield observed in the current study, in broilers consuming alkaline protease-supplemented diets, may be due to improved nutrient absorption, including proteins, AAs, vitamins, and minerals, essential for muscle development. A variety of factors, primarily the improved digestion of protein and availability of AAs via the action of exogenous alkaline protease, are responsible for this improvement. This increased uptake of essential AAs ensures muscle development instead of fat deposition, leading to higher BY and decreased abdominal fat deposition in broilers.

The gut health of broilers is determined by various factors, including structural growth. Taller villi, with an increased surface area, can increase the uptake of available nutrients. Newly formed epithelial cells from the intestinal mucosal crypts move along with the villi to the top. An increased crypt depth indicates slower cell changes and suggests that the gut can redirect more energy for effective nutrient absorption, thereby improving overall feeding and growth efficiency. The supplementation of alkaline protease improved intestinal morphology, demonstrated by an improved duodenal VH and VCR and reduced CD in broilers relative to the control diet, which led to improved nutrient digestibility. The improved intestinal morphology in the present study related to gut health aligns with expected outcomes and the literature on broilers [37]. Hussain et al. (2019) reported a 3.5% improved VH, 3% lower CD, and 6.3% better VCR in broilers fed alkaline protease-supplemented diets [35].

The improved AIDP and AIDAA via protease supplementation, compared to those on control diets in the current research trial, are consistent with the available literature on broilers [29]. These later researchers reported an improved digestibility of protein by up to 6.6% and of AA from 3 to 10.5% in broilers fed alkaline protease-supplemented diets [19]. Vieira et al. (2013) found an improved digestibility of CP up to 2.7%, and AIDAA from 0.1 up to 8% in broilers fed diets supplemented with alkaline protease relative to those on the control diet [9]. Qiu et al. (2023) observed an improvement of 5.4% in CP and from 1.7 to 7.8% in AA digestibility in broilers fed an alkaline protease-supplemented diet in comparison with those consuming a control diet [30]. Cowieson et al. (2016), likewise, reported an increased AIDAA of up to 3.5% by supplementing alkaline protease in the diet of broilers [12]. This improved digestibility of protein and AAs via protease supplementation may be due to the improved activity of the pancreas in the gut of broilers. The increased function of the pancreas facilitates the sufficient secretion of enzymes, which, along with exogenous protease, resulted in improved nutrient digestibility and the digestibility of almost all AAs up to 3.7% [28]. Bertechini et al. (2020) reported an improved AIDAA by up to 5.5% in broilers fed alkaline protease-supplemented PBM-based diets compared with the other diets [38]. This variation in findings might be associated with differences in protease pH, source, and dietary supplementation level. The present study provides significant results on the impact of protease sources on nutrient digestibility in broilers consuming a PBM-based diet. The inclusion of protease resulted in a notable improvement in DM digestibility, showing the highest improvement in broilers fed alkaline protease-supplemented PBM-based diets. This may be due to the ability of alkaline proteases to hydrolyze complex proteins into AAs, making other nutrients more accessible for digestion and absorption. Improved DM digestibility and increased nutrient availability in broilers lead to improved feed efficiency and overall growth performance [12].

The observed findings on the improved cecal microbiota count after protease supplementation are consistent with the available literature on broilers [39,40,41]. Gut microbiota are crucial for improving feed efficiency and/or nutrient digestibility [40]. Prior investigators have observed that protease supplementation may contribute to a shift in the substrates available in the intestine for bacterial growth [41]. The selection of *Salmonella*, *E. coli*, *Clostridia*, and *Lactobacillus* was based on their established relevance to poultry gut health, as well as their documented responses to dietary interventions, including protease supplementation. *Salmonella* and *E. coli* are common enteric zoonotic pathogens that cause both animal and public health risks [42]. *Clostridia* is the pathogen responsible for necrotic enteritis, a major intestinal disease in broilers [43]. *Lactobacillus*, on the other hand, is an important microbe involved in maintaining the integrity of the gut barrier and exclusion of the pathogen [44]. These microbiota were selected based on their frequent use as microbial indicators in nutritional and microbiota studies [39,45]. Rao et al. (2023) observed an increased cecal *Lactobacillus* and reduced *E. coli* population after dietary protease supplementation in broilers [39]. The improvement in the health-beneficial microbial population via protease supplementation might be related to the availability of certain Aas, such as phenylalanine, glutamine, proline, and glutamate, which have a beneficial influence on the growth of *Lactobacillus* spp. in the intestine [45]. This high efficacy of alkaline protease may be due to its higher activity in the slightly alkaline environment of the intestines, enhancing protein digestion and reducing the availability of substrates, thus limiting the nutrients available for pathogenic bacteria. Ponomarova et al. (2017) and Farrokhi et al. (2021), likewise, observed a higher cecal *Lactobacillus* count with lower *E. coli* populations in broilers fed protease-supplemented diets [45,46]. Protease supplementation significantly reduced *Salmonella*, *E. coli*, and *Clostridia* levels compared to the control, which suggests that protease is more effective at reducing the number of pathogens [47]. The ability of alkaline protease to reduce *E. coli* counts may be linked to its efficacy in increasing nutrient uptake and digestion, thus limiting the nutrients available for pathogenic bacteria [28]. The hydrolysis of protein into AAs may create a competitive environment for beneficial bacteria like *Lactobacilli*, which can inhibit the growth of *E. coli* through competitive exclusion [48]. Alkaline protease appears to be beneficial in controlling pathogenic bacterial populations in the gut, such as *Clostridia*, *E. coli*, and *Salmonella*, possibly through enhanced protein digestion and reduced protein fermentation in the hindgut [49]. Alkaline protease stops *Clostridia* from making harmful chemicals, which may improve gut health and performance as a whole [50].

## 5. Conclusions

In conclusion, the dietary supplementation of protease showed a healthy gut, with a more health-beneficial cecal microbiota profile with better nutrient apparent ileal digestibility, including AAs, resulting in better carcass traits and improved zootechnical performance in broilers fed PBM-based diets. Economically, protease supplementation presents a useful solution for reducing feed costs through efficient protein utilization and enabling the use of more affordable, non-conventional protein sources such as PBM, without compromising the production performance. The broilers fed the diet containing alkaline protease showed superior performance compared to the control and acidic and neutral protease-supplemented diets. Alkaline protease, therefore, can be used to augment the production performance of broilers fed diets that could increase the net profit of the producer and guarantee the accessibility of sustainable superior quality animal protein for the end users. Further research is warranted to explore its long-term effects, optimal inclusion rates, and synergistic use with other feed additives in diverse diet formulations.

## Figures and Tables

**Table 1:** Ingredient composition and nutrient analysis of the control diet ^(1)^ for broilers.

Ingredients (%)	Starter (1 to 10 d)	Grower (11 to 24 d)	Finisher (25 to 35 d)
Maize grain	52.61	58.8	64.07
Rice polishing	0.26	0.20	0.20
Canola oil	3.25	2.94	2.04
Soybean meal—44	29.5	23.73	21.0
Corn gluten meal—60	4.00	4.00	3.00
Canola meal—38	3.10	3.00	3.00
Guar meal—42	1.00	1.60	1.20
Poultry by-product meal—57.4	3.00	3.00	3.00
L-lysine HCl	0.44	0.43	0.43
DL-methionine	0.25	0.24	0.23
L-threonine	0.18	0.17	0.16
Common salt	0.20	0.20	0.20
Mono calcium phosphate	0.09	0.05	0.05
Limestone	1.82	1.34	1.12
Soda	0.10	0.10	0.10
Choline	0.05	0.05	0.05
Phytase	0.01	0.01	0.01
Premix ^(a)^	0.14	0.14	0.14
Protease ^(b,c,d)^	-	-	-
Celite ^(e)^	-	-	-
Total	100	100	100
Calculated composition, %, unless noted			
CP	23.5	22	20
ME (Kcal/kg)	3010	3075	3130
CF	3.64	3.47	3.32
EE	5.64	5.4	4.5
Dig. Lys	1.32	1.18	1.08
Dig. Meth	0.55	0.51	0.48
Dig. M+C	1.00	0.92	0.86
Dig. Thr	0.88	0.79	0.72
Analyzed composition (%)			
CP	23.1	21.7	19.6
CF	3.59	3.45	3.29
EE	5.63	5.36	4.47
Lys	1.30	1.17	1.07
Meth	0.6	0.56	0.51
Thr	0.86	0.77	0.69

^(a)^ Each kg of premix provided per kg of diet: vitamin A, 15,000 IU; vitamin D_3_, 3000 IU; vitamin E, 80 mg; vitamin K_3_, 3 mg; vitamin B_1_, 3 mg; vitamin B_2_, 8 mg; vitamin B_3_, 60 mg; vitamin B_5_, 15 mg, vitamin B_6_, 4 mg; vitamin B_9_, 2 mg; vitamin B_12_, 0.02 mg; vitamin H, 0.2 mg/Kg; choline chloride (60%), 700 mg; Zn (as ZnSO_4_.H_2_O), 80 mg; Cu (CuSO_4_.H_2_O), 10 mg; Fe (FeSO_4_.H_2_O), 60 mg; Mn (MnSO_4_.H_2_O), 80 mg; I (KI), 1 mg; Se, 0.2 mg; anti-oxidant mixture, 125 mg; Coxiril, 0.1 g/kg; enramycin, 0.1 g/kg; ^(b)^ acidic protease; ^(c)^ alkaline protease; ^(d)^ neutral protease; ^(e)^ Celite^®^; and source of acid insoluble ash (AIA). ^(1)^ T_0_, control: without protease supplementation; T_1_, acidic protease at 100 g/ton (50,000 U/g); T_2_, alkaline protease at 200 g/ton (25,000 U/g); and T_3_, neutral protease 200 g/ton (25,000 U/g).

**Table 2:** Effects of protease sources on growth parameters of the broilers ^(1)^ fed PBM based diets ^(2)^ from 1 to 35 d of age.

Parameter	Treatments	Age (d)			
		Starter (1–10)	Grower (11–24)	Finisher (25–35)	Overall (1–35)
FI (g/bird/d) ^(4)^	T_0_	31.6	86.7	131.4	85.0
	T_1_	31.5	86.8	128.2	84.0
	T_2_	31.5	86.4	129.5	84.3
	T_3_	31.3	86.5	128.5	83.9
	SEM ^(3)^	0.16	0.14	1.01	0.34
	*p*-value	0.56	0.12	0.13	0.13
BWG (g/bird/d) ^(5)^	T_0_	28.5 ^c^	61.7 ^d^	77.6	57.2 ^c^
	T_1_	29.7 ^b^	62.3 ^c^	78.1	58.0 ^bc^
	T_2_	30.5 ^a^	65.3 ^a^	79.6	59.9 ^a^
	T_3_	29.7 ^b^	64.2 ^b^	78.7	58.9 ^b^
	SEM ^(3)^	0.12	0.17	0.75	0.24
	*p*-value	0.01	0.01	0.30	0.01
FCR ^(6)^	T_0_	1.11 ^c^	1.42 ^d^	1.69 ^b^	1.49 ^c^
	T_1_	1.06 ^b^	1.39 ^c^	1.64 ^a^	1.45 ^b^
	T_2_	1.03 ^a^	1.32 ^a^	1.63 ^a^	1.41 ^a^
	T_3_	1.05 ^ab^	1.35 ^b^	1.63 ^a^	1.43 ^a^
	SEM ^(3)^	0.01	0.005	0.011	0.005
	*p*-value	0.01	0.01	0.01	0.01

^a–d^ Means without a common superscript within a column significantly (*p* < 0.05) differ. ^(1)^ Each value represents the mean of 8 replicates (3 birds per replicate). ^(2)^ T_0_, control: without protease supplementation; T_1_, acidic protease at 100 g/ton (50,000 U/g); T_2_, alkaline protease at 200 g/ton (25,000 U/g); and T_3_, neutral protease 200 g/ton (25,000 U/g). ^(3)^ SEM, standard error mean. ^(4)^ FI (g/bird/d), feed intake (gram per bird per day); ^(5)^ BWG (g/bird/d), body weight gain (gram per bird per day); and ^(6)^ FCR, feed conversion ratio.

**Table 3:** Effects of protease sources on carcass characteristics in the broilers ^(1)^ fed PBM-based diets ^(2)^.

Item	(%)				
	LW ^(3)^ (g)	DWG ^(3)^	DWWG ^(3)^	BY ^(3)^	LQ ^(3)^
T_0_	2042 ^c^	67.8 ^d^	62.4 ^d^	20.9 ^c^	9.33 ^c^
T_1_	2068 ^bc^	68.8 ^c^	63.6 ^c^	21.4 ^c^	9.46 ^b^
T_2_	2135 ^a^	71.6 ^a^	66.2 ^a^	23.6 ^a^	9.66 ^a^
T_3_	2102 ^b^	70.9 ^b^	64.5 ^b^	22.4 ^b^	9.52 ^b^
SEM ^(4)^	8.25	0.25	0.23	0.18	0.02
*p*-value	<0.001	<0.001	<0.001	<0.001	<0.001

^a–d^ Means without a common superscript within a column significantly (*p* < 0.05) differ. ^(1)^ Each value represents the mean of 8 replicates (3 birds per replicate). ^(2)^ T_0_, control: without protease supplementation; T_1_, acidic protease at 100 g/ton (50,000 U/g); T_2_, alkaline protease at 200 g/ton (25,000 U/g); and T_3_, neutral protease 200 g/ton (25,000 U/g). ^(3)^ LW, live weight; DWG, dressed weight with giblets; DWWG, dressed weight without giblets; BY, breast yield; and LQY, leg quarter yield. ^(4)^ SEM, standard error mean.

**Table 4:** Effects of protease sources on villus height (µm), crypt depth (µm), and villus height to crypt depth ratio (VCR) in the duodenum of broilers ^(1)^ fed PBM-based diets ^(2)^.

Item	VH (μm)	CD (μm)	VCR
T_0_	1564 ^c^	297 ^c^	5.27 ^c^
T_1_	1697 ^b^	276 ^b^	6.14 ^b^
T_2_	1735 ^a^	268 ^a^	6.47 ^a^
T_3_	1707 ^b^	276 ^b^	6.17 ^b^
SEM ^(3)^	8.92	1.67	0.05
*p*-value	<0.001	<0.001	<0.001

^a–c^ Means without a common superscript within a column significantly (*p* < 0.05) differ. ^(1)^ Each value represents the mean of 8 replicates (3 birds per replicate). ^(2)^ T_0_, control: without protease supplementation; T_1_, acidic protease at 100 g/ton (50,000 U/g); T_2_, alkaline protease at 200 g/ton (25,000 U/g); and T_3_, neutral protease 200 g/ton (25,000 U/g). ^(3)^ SEM, standard error mean.

**Table 5:** Effects of protease sources on apparent ileal digestibility of crude protein, amino acids, and dry matter in the broilers ^(1)^ fed PBM-based diets ^(2)^.

Item (%)	T_0_	T_1_	T_2_	T_3_	SEM ^(3)^	*p*-Value
DM	70.5 ^c^	72.8 ^b^	75.2 ^a^	73.1 ^b^	0.70	0.01
CP	78.5 ^c^	80.2 ^b^	82.8 ^a^	80.9 ^b^	0.60	0.01
EAAs ^(4)^						
Meth	82.7 ^c^	85.1 ^b^	87.9 ^a^	85.8 ^b^	0.85	0.01
Lys	84.3 ^c^	86.7 ^b^	89.2 ^a^	87.4 ^b^	0.70	0.01
Leu	80.3 ^c^	82.5 ^b^	85.4 ^a^	83.2 ^b^	0.60	0.01
Ile	73.2 ^b^	75.0 ^ab^	77.8 ^a^	75.6 ^ab^	0.55	0.01
Thr	64.5 ^c^	66.7 ^b^	69.4 ^a^	67.2 ^b^	0.70	0.01
Val	60.5 ^c^	62.2 ^b^	64.9 ^a^	63.1 ^b^	0.55	0.01
His	80.2 ^c^	82.3 ^b^	84.8 ^a^	82.9 ^b^	0.60	0.01
Arg	85.4 ^c^	87.6 ^b^	90.3 ^a^	88.4 ^b^	0.60	0.01
Phe	75.7 ^c^	77.8 ^b^	80.5 ^a^	78.3 ^b^	0.65	0.01
NEAAs ^(5)^						
Gly	63.5 ^c^	65.4 ^b^	68.3 ^a^	66.1 ^b^	0.60	0.01
Ala	72.3 ^b^	74.6 ^ab^	76.9 ^a^	75.1 ^ab^	0.60	0.01
Cys	78.5 ^c^	80.6 ^b^	84.2 ^a^	82.1 ^b^	0.90	0.01
Ser	73.5 ^c^	75.2 ^b^	77.6 ^a^	75.8 ^b^	0.70	0.01
Glu	83.7 ^b^	85.3 ^ab^	87.1 ^a^	85.9 ^ab^	0.40	0.01
Asp	70.6 ^c^	72.4 ^b^	75.6 ^a^	73.2 ^b^	0.70	0.01
Tyr	78.3 ^c^	80.7 ^b^	84.1 ^a^	81.6 ^b^	0.90	0.01
Orn	75.1 ^a^	75.8 ^a^	77.1 ^a^	76.5 ^a^	0.50	0.15
Pro	74.8 ^c^	76.7 ^b^	79.4 ^a^	77.2 ^ab^	0.75	0.01

^a–c^ Means without a common superscript within a column significantly (*p* < 0.05) differ. ^(1)^ Each value represents the mean of 8 replicates (3 birds per replicate). ^(2)^ T_0_, control: without protease supplementation; T_1_, acidic protease at 100 g/ton (50,000 U/g); T_2_, alkaline protease at 200 g/ton (25,000 U/g); and T_3_, neutral protease 200 g/ton (25,000 U/g). ^(3)^ SEM, standard error mean. ^(4)^ EAAs, essential amino acids; Meth, methionine; Lys, lysine; Leu, leucine; Ile, isoleucine; Thr, threonine; Val, valine; His, histidine; Arg, arginine; and Phe, phenylalanine. ^(5)^ NEAAs, non-essential amino acids; Gly, glycine; Ala, alanine; Asp, aspartic acid; Cys, cysteic acid; Ser, serine; Glu, glutamic acid; Tyr, tyrosine; Orn, ornithine; and Pro, proline.

**Table 6:** Effects of protease sources on the counts (log cfu/g) of mucosa-associated bacteria in the cecum of broilers ^(1)^ fed PBM-based diets ^(2)^.

Item	*Salmonella*	*E. coli*	*Clostridia*	*Lactobacilli*
T_0_	4.13 ^a^	8.09 ^a^	3.80 ^a^	7.20 ^c^
T_1_	3.30 ^b^	5.82 ^b^	3.55 ^b^	7.52 ^b^
T_2_	3.15 ^c^	5.02 ^c^	3.06 ^c^	8.53 ^a^
T_3_	3.18 ^c^	5.11 ^c^	3.46 ^b^	7.82 ^b^
SEM ^(3)^	0.11	0.32	0.07	0.14
*p*-value	<0.001	<0.001	<0.001	<0.001

^a–c^ Means without a common superscript within a column significantly (*p* < 0.05) differ. ^(1)^ Each value represents the mean of 8 replicates (3 birds per replicate). ^(2)^ T_0_, control: without protease supplementation; T_1_, acidic protease at 100 g/ton (50,000 U/g); T_2_, alkaline protease at 200 g/ton (25,000 U/g); and T_3_, neutral protease 200 g/ton (25,000 U/g). ^(3)^ SEM, standard error mean.

## Data Availability

The original contributions presented in this study are included in the article. Further inquiries can be directed to the corresponding author.
